# Some Spinal Cord Lesions

**Published:** 1911-03-04

**Authors:** 


### Neurology.

### I.—SOME SPINAL CORD LESIONS.

### PRIMARY SPASTIC AND ATAXIC PARAPLEGIA.

#### Primary Lateral Sclerosis.

This is a chronic disease characterised clinically by gradual loss of power in the legs with spastic rigidity and exaggerated reflexes. The lesion, as far as it is known at present, is a chronic primary degeneration of the pyramidal tracts of the spinal cord. Males between the ages of twenty and forty are more commonly affected than females. In the majority of cases there is no apparent cause. Occasionally it follows concussion of the spine and exposure to cold and wet, and syphilis is a predisposing factor.

#### Onset.

The onset is slow and insidious. The first indications of the disease, as a rule, are a feeling of weakness and stiffness in the legs, which comes on after exercise, the patient finding that he tires more readily after an ordinary walk than formerly, and he gradually notices that the distance he is able to go becomes less and less. The feeling of stiffness is also experienced on first getting out of bed in the morning. In a well-developed case the legs are extremely rigid in a position of extension and adduction. The rigidity may be constant. If, however, the muscles of the legs are flaccid, the slightest touch or movement may cause them to contract and become rigid. The loss of power varies. In some cases it is well marked, whereas in others even when rigidity is well established the loss of power may be slight. On account of the spasm of the adductor muscles of the thigh, the knees are kept close together, and it may be impossible to separate the legs. The gait is characteristic: the legs are stiff, kept close together and moved from the hips with a rotatory or swinging movement of the body. The toes drag along the ground, and when the foot touches the floor a clonus may be produced. In some cases the spasm of the adductors is so excessive that the legs are crossed—scissor gait. The arms are generally unaffected.

#### Sensory Symptoms.

Sensory symptoms are usually absent. Some patients experience slight pains in the back and legs, but there is no anæsthesia. The knee-jerks are exaggerated. Rectus and ankle clonus are readily elicited. The superficial reflexes are increased, and the plantar reflex is extensor in character. In some cases the slightest touch may produce violent clonic spasm of the legs.

The muscles are, as a rule, unaffected as regard their nutrition. The calf muscles may, however, be slightly hypertrophied; in some cases very slight wasting may be noticed.

The muscles react normally to both the interrupted and the continuous currents. The functions of the bladder and rectum are usually unimpaired until quite late in the disease. Slight nystagmus is present in a small proportion of the cases.

The course of the disease is very chronic. It may reach a certain stage and remain stationary for many years. The chief indications which point to a diagnosis of primary spastic paraplegia are the gradual onset of the disease in the legs without any apparent cause and the absence of sensory symptoms, muscular atrophy, or impairment of the functions of the bladder and rectum. The presence of pain and loss of sensation would point to a focal lesion of the cord involving the posterior nerve roots; of atrophy of the muscles of the hands to amyotrophic lateral sclerosis; of inco-ordination and unsteadiness when standing with the eyes closed and the heels close together to ataxic paraplegia; and of loss of sensation to heat, cold and pain, to syringomyelia.

#### Ataxic Paraplegia.

This is a chronic disease of the spinal cord characterised clinically by the association of ataxy with spastic paraplegia. The lesion is a combined sclerosis of the posterior and lateral columns of the cord; the posterior columns are mostly affected in the dorsal region, whereas in the lumbar region they are usually unaffected. The sclerosis does not always reach to the posterior edge of the cord; the anterior four-fifths is the part most frequently involved. Neither the posterior root zones nor the posterior nerve roots are affected. The crossed pyramidal tracts are usually sclerosed throughout the cord. The sclerosis of the tracts explains the loss of power and the rigidity of the legs; the sclerosis of the posterior columns accounts for the ataxy; whilst the increased reflexes are explained by the sclerosis of the pyramidal tracts and the absence of morbid changes in the posterior root zones and posterior nerve roots.

#### Incidence.

The malady usually attacks males between the ages of thirty and forty. It is said that it may follow exposure to cold, severe exertion, concussion of the spine, and great sexual excess. The onset is slow and gradual. The patient first notices that he readily becomes tired after taking an ordinary amount of exercise, and there may be slight weakness in the legs. The arms may be similarly affected. Next a difficulty in turning round quickly and unsteadiness when walking in the dark may be experienced. A dull, heavy pain in the lower part of the back and sacral region is another early symptom. In a well-marked case there will be noticed weakness of the legs, rigidity, unsteady, ataxic gait, a difficulty in turning round quickly when walking, and an inability to stand still with the eyes shut and the heels close together. The arms are weak and their movements inco-ordinated in some cases. Tremulous movements of the lips and facial muscles may be present.

Symptoms.

Anæsthesia is unusual; in some cases, however, slight anæsthesia of the legs and trunk may be noticed. A dull pain in the legs after exertion and a dull, heavy pain over the sacrum may be early symptoms. Hyperæsthesia is rare. Lightning pains are absent. The knee-jerks are increased, and ankle and rectus clonus may be present. The plantar reflex is extensor. The abdominal reflex is generally absent, and the cremasteric reflex may be absent also. If the arms are affected, well-marked radial and ulnar wrist-jerks and tricipital and bicipital elbow-jerks may be obtained.

There are no marked trophic changes, though in advanced cases there may be some wasting of the muscles as a result of disuse. The electrical reactions are normal. Sexual power is frequently lost in the early stages of the disease. Inability to empty the bladder with subsequent habitual distension is sometimes noticed quite early, whereas in other cases the bladder and rectum functions are not impaired until quite late in the disease. The pupils react to light and to accommodation. Nystagmus may be present in some cases. The special senses are otherwise unaffected.

Diagnosis.

The course of the disease is chronic. The diagnosis from primary spastic paraplegia depends on the presence of inco-ordination. In the early stages, when inco-ordination is the predominant symptom, the case may be mistaken for one of locomotor ataxy, but is distinguished from this disease by the presence of the exaggerated knee-jerks and the absence of crises, lightning pains, and Argyll-Robertson pupils. A tumour in the cerebellum pressing on the pyramidal fibres in the pons may cause inco-ordination and weakness of the legs; but vomiting, occipital headache, and optic neuritis are present in addition, and serve to distinguish such a condition from ataxic paraplegia. Disseminated sclerosis is more difficult to exclude sometimes, but the intention tremors of this disease are not found in ataxic paraplegia, nor are the scotomata that are so common in disseminated sclerosis. Speech helps little, for the "scanning" speech of the latter is very seldom met with in any marked degree. Pronounced nystagmus would be against ataxic paraplegia and in favour of disseminated sclerosis if cerebral tumour can be excluded.

